# Ethyl 2-(3-acetyl-6-methyl-2-oxo-2*H*-pyran-4-yl­oxy)acetate

**DOI:** 10.1107/S1600536810001601

**Published:** 2010-01-20

**Authors:** Muhammad Rabnawaz, Stacy D. Benson, Burhan Khan, Muhammad Raza Shah

**Affiliations:** a H.E.J. Research Institute of Chemistry, International Center for Chemical and Biological Sciences, University of Karachi, Karachi 75270, Pakistan; b Oklahoma State University, Department of Chemistry, 107 Physical Sciences, Stillwater, OK 74078-3071, USA

## Abstract

The title compound, C_12_H_14_O_6_, features a roughly planar mol­ecule (r.m.s. deviation for all non-H atoms = 0.287 Å). In the crystal, the mol­ecules are held together by C—H⋯O hydrogen bonds.

## Related literature

For the use of dehydro­acetic acid as a starting material in the synthesis of heterocyclic ring systems, see: Prakash *et al.* (2004 ▶), and of biologically important mol­ecules such as coumarins, see: Hernandez-Galan *et al.* (1993 ▶).
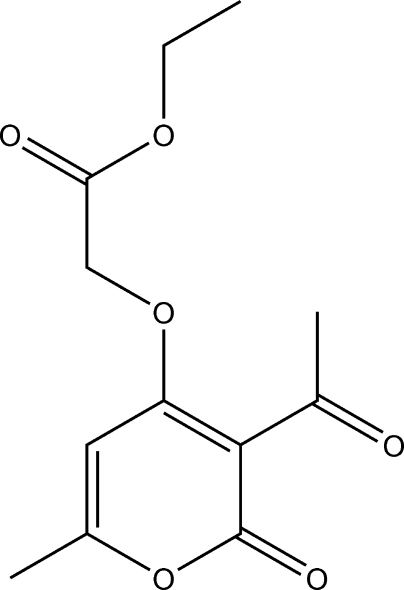


## Experimental

### 

#### Crystal data

C_12_H_14_O_6_
*M* *_r_* = 254.23
Triclinic, 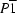

*a* = 7.8258 (10) Å
*b* = 8.2722 (11) Å
*c* = 10.0838 (13) Å
α = 77.374 (7)°
β = 77.759 (6)°
γ = 88.857 (7)°
*V* = 622.28 (14) Å^3^
*Z* = 2
Mo *K*α radiation
μ = 0.11 mm^−1^
*T* = 298 K
0.72 × 0.13 × 0.11 mm

#### Data collection

Bruker SMART APEXII diffractometer
Absorption correction: multi-scan (*SADABS*; Bruker, 2001 ▶) *T*_min_ = 0.925, *T*_max_ = 0.988
14279 measured reflections
3039 independent reflections
2330 reflections with *I* > 2σ(*I*)
*R*_int_ = 0.036

#### Refinement

*R*[*F* ^2^ > 2σ(*F* ^2^)] = 0.050
*wR*(*F* ^2^) = 0.159
*S* = 1.04
3039 reflections
166 parameters
H-atom parameters constrained
Δρ_max_ = 0.35 e Å^−3^
Δρ_min_ = −0.20 e Å^−3^

### 

Data collection: *APEX2* (Bruker, 2008 ▶); cell refinement: *SAINT-Plus* (Bruker, 2008 ▶); data reduction: *SAINT-Plus*; program(s) used to solve structure: *SHELXS97* (Sheldrick, 2008 ▶); program(s) used to refine structure: *SHELXL97* (Sheldrick, 2008 ▶); molecular graphics: *XP* in *SHELXTL* (Sheldrick, 2008 ▶); software used to prepare material for publication: *SHELXL97*.

## Supplementary Material

Crystal structure: contains datablocks I, global. DOI: 10.1107/S1600536810001601/bt5170sup1.cif

Structure factors: contains datablocks I. DOI: 10.1107/S1600536810001601/bt5170Isup2.hkl

Additional supplementary materials:  crystallographic information; 3D view; checkCIF report

## Figures and Tables

**Table 1:** Hydrogen-bond geometry (Å, °)

*D*—H⋯*A*	*D*—H	H⋯*A*	*D*⋯*A*	*D*—H⋯*A*
C6*A*—H6*A*1⋯O2^i^	0.96	2.53	3.462 (2)	165
C5—H5⋯O3*A*^i^	0.93	2.38	3.3053 (19)	174
C2*A*—H2*A*1⋯O3*A*^i^	0.97	2.57	3.355 (2)	138
C2*E*—H2*E*2⋯O1^ii^	0.96	2.54	3.484 (3)	169

Symmetry codes: (i) 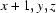; (ii) 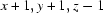.

**Fig. 1.**
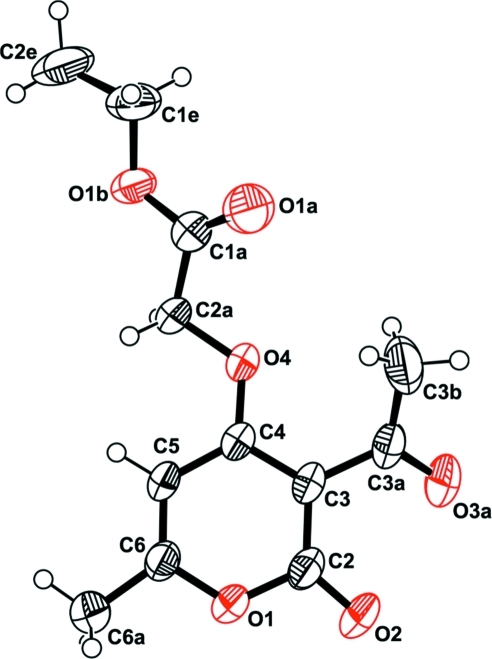
Crystal Structure of Ethyl 2-(3-acetyl-6-methyl-2-oxo-2H-pyran-4-yloxy) acetate (50% ellipsoids).

## supplementary crystallographic information

### Comment

3-Acetyl-4-hydroxy-6-methyl-2-oxo-2*H*-pyran (dehydroacetic acid) is a versatile starting material for the synthesis of a wide variety of heterocyclic ring systems (Prakash *et al.*, 2004) and biologically important molecules like coumarins (Hernandez-Galan *et al.*, 1993).

### Experimental

The dehydroacetic acid (500 mg, 3 mmol) was treated with ethylbromoacetate (2 g, 12 mmol) in acetone in the presence of K_2_CO_3_ (1.6 g, 12 mmol). The reaction mixture was refluxed for 3 h monitored with TLC at regular intervals of 30 minutes. The reaction was quenched by addition of 1 N HCl (10 ml) and the aqueous layer was extracted with ethyl acetate three times. The combined organic layers were concentrated under reduced pressure. The crude residue was dissolved in hot ethanol. The slow evaporation of ethanol yielded colorless needle-like crystals (90%, 680 mg).

### Refinement

The H atoms were placed in calculated positions and allowed to ride on their carrier atoms with C—H = 0.93–0.96 Å and with *U*_iso_ = 1.2*U*_eq_(C) for CH and CH_2_ and *U*_iso_ = 1.5*U*_eq_(C) for CH_3_ groups.

### Crystal data

**Table:**

C_12_H_14_O_6_	*Z* = 2
*M**_r_* = 254.23	*F*(000) = 268
Triclinic, *P*1	*D*_x_ = 1.357 Mg m^−^^3^
Hall symbol: -P 1	Mo *K*α radiation, λ = 0.71073 Å
*a* = 7.8258 (10) Å	Cell parameters from 6664 reflections
*b* = 8.2722 (11) Å	θ = 2.1–28.3°
*c* = 10.0838 (13) Å	µ = 0.11 mm^−^^1^
α = 77.374 (7)°	*T* = 298 K
β = 77.759 (6)°	Rectangular prism, clear colourless
γ = 88.857 (7)°	0.72 × 0.13 × 0.11 mm
*V* = 622.28 (14) Å^3^	

### Data collection

**Table:**

Bruker SMART APEXII diffractometer	3039 independent reflections
Radiation source: fine-focus sealed tube	2330 reflections with *I* > 2σ(*I*)
graphite	*R*_int_ = 0.036
Detector resolution: 83.33 pixels mm^-1^	θ_max_ = 28.3°, θ_min_ = 2.1°
φ scans and ω scans with κ offsets	*h* = −10→10
Absorption correction: multi-scan (*SADABS*; Bruker, 2001)	*k* = −11→10
*T*_min_ = 0.925, *T*_max_ = 0.988	*l* = −13→13
14279 measured reflections	

### Refinement

**Table:**

Refinement on *F*^2^	Primary atom site location: structure-invariant direct methods
Least-squares matrix: full	Secondary atom site location: difference Fourier map
*R*[*F*^2^ > 2σ(*F*^2^)] = 0.050	Hydrogen site location: inferred from neighbouring sites
*wR*(*F*^2^) = 0.159	H-atom parameters constrained
*S* = 1.04	*w* = 1/[σ^2^(*F*_o_^2^) + (0.0773*P*)^2^ + 0.1512*P*] where *P* = (*F*_o_^2^ + 2*F*_c_^2^)/3
3039 reflections	(Δ/σ)_max_ < 0.001
166 parameters	Δρ_max_ = 0.35 e Å^−^^3^
0 restraints	Δρ_min_ = −0.20 e Å^−^^3^

### Special details

**Table:**

Geometry. All e.s.d.'s (except the e.s.d. in the dihedral angle between two l.s. planes) are estimated using the full covariance matrix. The cell e.s.d.'s are taken into account individually in the estimation of e.s.d.'s in distances, angles and torsion angles; correlations between e.s.d.'s in cell parameters are only used when they are defined by crystal symmetry. An approximate (isotropic) treatment of cell e.s.d.'s is used for estimating e.s.d.'s involving l.s. planes.
Refinement. Refinement of *F*^2^ against ALL reflections. The weighted *R*-factor *wR* and goodness of fit *S* are based on *F*^2^, conventional *R*-factors *R* are based on *F*, with *F* set to zero for negative *F*^2^. The threshold expression of *F*^2^ > 2σ(*F*^2^) is used only for calculating *R*-factors(gt) *etc*. and is not relevant to the choice of reflections for refinement. *R*-factors based on *F*^2^ are statistically about twice as large as those based on *F*, and *R*- factors based on ALL data will be even larger.

### Fractional atomic coordinates and isotropic or equivalent isotropic displacement parameters (Å^2^)

**Table:**

	*x*	*y*	*z*	*U*_iso_*/*U*_eq_	
O1	0.57091 (13)	0.60585 (14)	1.11130 (11)	0.0528 (3)	
C2	0.44227 (18)	0.6846 (2)	1.04378 (17)	0.0494 (4)	
C3	0.50118 (17)	0.81700 (18)	0.92489 (15)	0.0438 (3)	
C4	0.67613 (17)	0.86621 (18)	0.89085 (15)	0.0419 (3)	
C5	0.79728 (17)	0.78287 (18)	0.96663 (16)	0.0445 (3)	
H5	0.9142	0.8175	0.9433	0.053*	
C6	0.74139 (18)	0.65385 (19)	1.07210 (15)	0.0453 (3)	
C6A	0.8505 (2)	0.5494 (2)	1.15859 (19)	0.0626 (5)	
H6A1	0.9708	0.5851	1.1248	0.094*	
H6A2	0.8379	0.4358	1.1537	0.094*	
H6A3	0.8134	0.5597	1.2534	0.094*	
C3A	0.36798 (19)	0.8935 (2)	0.84701 (18)	0.0525 (4)	
C3B	0.4174 (3)	0.9705 (5)	0.6972 (3)	0.1171 (12)	
H3B1	0.3199	0.9630	0.6548	0.176*	
H3B2	0.5145	0.9138	0.6542	0.176*	
H3B3	0.4499	1.0849	0.6854	0.176*	
O3A	0.21609 (15)	0.8898 (2)	0.90547 (17)	0.0803 (5)	
O4	0.72493 (13)	0.99457 (14)	0.78399 (13)	0.0576 (3)	
C1A	0.9346 (2)	1.1577 (2)	0.60243 (17)	0.0525 (4)	
C2A	0.8981 (2)	1.0641 (2)	0.74992 (18)	0.0562 (4)	
H2A1	0.9816	0.9768	0.7622	0.067*	
H2A2	0.9088	1.1378	0.8106	0.067*	
O2	0.29702 (14)	0.62711 (18)	1.09368 (15)	0.0704 (4)	
O1A	0.8501 (2)	1.1503 (2)	0.51873 (16)	0.0947 (6)	
O1B	1.08032 (15)	1.24797 (15)	0.57701 (11)	0.0587 (3)	
C1E	1.1521 (3)	1.3319 (3)	0.43439 (19)	0.0748 (6)	
H1E1	1.0694	1.4101	0.3996	0.090*	
H1E2	1.1769	1.2523	0.3755	0.090*	
C2E	1.3156 (3)	1.4200 (4)	0.4343 (3)	0.1057 (9)	
H2E1	1.2882	1.5037	0.4875	0.159*	
H2E2	1.3712	1.4710	0.3404	0.159*	
H2E3	1.3930	1.3424	0.4747	0.159*	

### Atomic displacement parameters (Å^2^)

**Table:**

	*U*^11^	*U*^22^	*U*^33^	*U*^12^	*U*^13^	*U*^23^
O1	0.0358 (5)	0.0632 (7)	0.0518 (6)	−0.0105 (5)	−0.0048 (4)	0.0003 (5)
C2	0.0325 (7)	0.0602 (9)	0.0533 (8)	−0.0079 (6)	−0.0035 (6)	−0.0126 (7)
C3	0.0282 (6)	0.0524 (8)	0.0514 (8)	−0.0040 (6)	−0.0069 (6)	−0.0141 (6)
C4	0.0306 (6)	0.0461 (7)	0.0475 (7)	−0.0052 (5)	−0.0062 (5)	−0.0084 (6)
C5	0.0280 (6)	0.0528 (8)	0.0512 (8)	−0.0071 (5)	−0.0080 (5)	−0.0077 (6)
C6	0.0338 (7)	0.0543 (8)	0.0469 (7)	−0.0054 (6)	−0.0076 (6)	−0.0096 (6)
C6A	0.0500 (9)	0.0741 (11)	0.0578 (10)	−0.0051 (8)	−0.0168 (7)	0.0039 (8)
C3A	0.0312 (7)	0.0618 (9)	0.0665 (10)	−0.0022 (6)	−0.0129 (6)	−0.0156 (8)
C3B	0.0486 (11)	0.212 (3)	0.0718 (14)	0.0101 (15)	−0.0214 (10)	0.0165 (17)
O3A	0.0314 (6)	0.1083 (11)	0.0932 (10)	0.0034 (6)	−0.0130 (6)	−0.0053 (8)
O4	0.0332 (5)	0.0616 (7)	0.0687 (7)	−0.0110 (5)	−0.0157 (5)	0.0110 (5)
C1A	0.0461 (8)	0.0541 (9)	0.0544 (9)	−0.0057 (7)	−0.0108 (7)	−0.0052 (7)
C2A	0.0361 (7)	0.0655 (10)	0.0578 (9)	−0.0158 (7)	−0.0116 (6)	0.0090 (7)
O2	0.0346 (6)	0.0894 (9)	0.0752 (8)	−0.0196 (6)	−0.0017 (5)	−0.0007 (7)
O1A	0.0861 (11)	0.1290 (14)	0.0670 (9)	−0.0384 (10)	−0.0321 (8)	0.0022 (9)
O1B	0.0544 (7)	0.0653 (7)	0.0469 (6)	−0.0197 (5)	−0.0051 (5)	0.0040 (5)
C1E	0.0799 (13)	0.0853 (13)	0.0456 (9)	−0.0179 (10)	−0.0010 (9)	0.0044 (9)
C2E	0.0910 (17)	0.127 (2)	0.0702 (13)	−0.0493 (15)	0.0077 (12)	0.0197 (13)

### Geometric parameters (Å, °)

**Table:**

O1—C6	1.3501 (16)	C3B—H3B1	0.9600
O1—C2	1.404 (2)	C3B—H3B2	0.9600
C2—O2	1.2024 (17)	C3B—H3B3	0.9600
C2—C3	1.436 (2)	O4—C2A	1.4256 (17)
C3—C4	1.3856 (17)	C1A—O1A	1.189 (2)
C3—C3A	1.486 (2)	C1A—O1B	1.3228 (18)
C4—O4	1.3326 (18)	C1A—C2A	1.489 (2)
C4—C5	1.417 (2)	C2A—H2A1	0.9700
C5—C6	1.337 (2)	C2A—H2A2	0.9700
C5—H5	0.9300	O1B—C1E	1.450 (2)
C6—C6A	1.481 (2)	C1E—C2E	1.485 (3)
C6A—H6A1	0.9600	C1E—H1E1	0.9700
C6A—H6A2	0.9600	C1E—H1E2	0.9700
C6A—H6A3	0.9600	C2E—H2E1	0.9600
C3A—O3A	1.2073 (19)	C2E—H2E2	0.9600
C3A—C3B	1.476 (3)	C2E—H2E3	0.9600
			
C6—O1—C2	122.89 (12)	H3B1—C3B—H3B2	109.5
O2—C2—O1	113.96 (15)	C3A—C3B—H3B3	109.5
O2—C2—C3	129.34 (16)	H3B1—C3B—H3B3	109.5
O1—C2—C3	116.68 (12)	H3B2—C3B—H3B3	109.5
C4—C3—C2	118.59 (13)	C4—O4—C2A	121.02 (12)
C4—C3—C3A	124.32 (14)	O1A—C1A—O1B	124.77 (16)
C2—C3—C3A	117.09 (12)	O1A—C1A—C2A	126.07 (16)
O4—C4—C3	117.09 (13)	O1B—C1A—C2A	109.14 (14)
O4—C4—C5	121.74 (12)	O4—C2A—C1A	108.53 (13)
C3—C4—C5	121.17 (13)	O4—C2A—H2A1	110.0
C6—C5—C4	119.19 (12)	C1A—C2A—H2A1	110.0
C6—C5—H5	120.4	O4—C2A—H2A2	110.0
C4—C5—H5	120.4	C1A—C2A—H2A2	110.0
C5—C6—O1	121.31 (13)	H2A1—C2A—H2A2	108.4
C5—C6—C6A	126.34 (14)	C1A—O1B—C1E	117.97 (14)
O1—C6—C6A	112.35 (13)	O1B—C1E—C2E	107.09 (17)
C6—C6A—H6A1	109.5	O1B—C1E—H1E1	110.3
C6—C6A—H6A2	109.5	C2E—C1E—H1E1	110.3
H6A1—C6A—H6A2	109.5	O1B—C1E—H1E2	110.3
C6—C6A—H6A3	109.5	C2E—C1E—H1E2	110.3
H6A1—C6A—H6A3	109.5	H1E1—C1E—H1E2	108.6
H6A2—C6A—H6A3	109.5	C1E—C2E—H2E1	109.5
O3A—C3A—C3B	118.99 (17)	C1E—C2E—H2E2	109.5
O3A—C3A—C3	119.99 (16)	H2E1—C2E—H2E2	109.5
C3B—C3A—C3	120.99 (14)	C1E—C2E—H2E3	109.5
C3A—C3B—H3B1	109.5	H2E1—C2E—H2E3	109.5
C3A—C3B—H3B2	109.5	H2E2—C2E—H2E3	109.5
			
C6—O1—C2—O2	−179.01 (14)	C2—O1—C6—C5	1.2 (2)
C6—O1—C2—C3	2.6 (2)	C2—O1—C6—C6A	−179.19 (14)
O2—C2—C3—C4	177.25 (16)	C4—C3—C3A—O3A	−153.29 (17)
O1—C2—C3—C4	−4.6 (2)	C2—C3—C3A—O3A	26.1 (2)
O2—C2—C3—C3A	−2.2 (3)	C4—C3—C3A—C3B	28.5 (3)
O1—C2—C3—C3A	175.96 (13)	C2—C3—C3A—C3B	−152.2 (2)
C2—C3—C4—O4	−176.87 (13)	C3—C4—O4—C2A	173.81 (14)
C3A—C3—C4—O4	2.5 (2)	C5—C4—O4—C2A	−6.3 (2)
C2—C3—C4—C5	3.3 (2)	C4—O4—C2A—C1A	159.18 (14)
C3A—C3—C4—C5	−177.39 (14)	O1A—C1A—C2A—O4	−13.5 (3)
O4—C4—C5—C6	−179.39 (14)	O1B—C1A—C2A—O4	168.37 (13)
C3—C4—C5—C6	0.5 (2)	O1A—C1A—O1B—C1E	−6.2 (3)
C4—C5—C6—O1	−2.8 (2)	C2A—C1A—O1B—C1E	172.01 (16)
C4—C5—C6—C6A	177.66 (16)	C1A—O1B—C1E—C2E	−178.72 (19)

### Hydrogen-bond geometry (Å, °)

**Table:**

*D*—H···*A*	*D*—H	H···*A*	*D*···*A*	*D*—H···*A*
C6A—H6A1···O2^i^	0.96	2.53	3.462 (2)	165
C5—H5···O3A^i^	0.93	2.38	3.3053 (19)	174
C2A—H2A1···O3A^i^	0.97	2.57	3.355 (2)	138
C2E—H2E2···O1^ii^	0.96	2.54	3.484 (3)	169

Symmetry codes: (i) *x*+1, *y*, *z*; (ii) *x*+1, *y*+1, *z*−1.
